# Episodic spastic paraparesis successfully treated with unaided blood transfusions: a case report

**DOI:** 10.1186/s13104-016-1918-5

**Published:** 2016-02-25

**Authors:** Loku Pathirage Manoji Muditha Kumari Pathirage, Indunil Wijeweera, Chandrika Jayasinghe, Saumya Jayabahu, Hasith Ravinda Wickramasinghe

**Affiliations:** Department of Medicine, Faculty of Medicine, University of Peradeniya, Peradeniya, Sri Lanka; Neurology Unit, General Hospital – Kandy, Kandy, Sri Lanka; Department of Radiology, Teaching Hospital – Peradeniya, Peradeniya, Sri Lanka

**Keywords:** Thalassaemia, Haemoglobin E, Paraplegia, Extramedullary haemopoiesis

## Abstract

**Background:**

Extramedullary haemopoiesis is a common compensatory phenomenon in most haemolytic anaemias. However, spinal cord compression due to extramedullary spinal epidural haemopoiesis is an extremely rare complication of thalassemia. In such situation patients present with paraplegia with a sensory level. Usual treatment options are surgery and/or radiotherapy.

**Case presentation:**

Here we report a 27 year old Sri Lankan Muslim male with haemoglobin E-Beta thalassaemia presented with episodic spastic paraparesis when he was anaemic which was dramatically responded to blood transfusion therapy.

**Conclusion:**

Most of the reported cases with paraplegia have been treated with surgery with or without radiation therapy or radiation therapy alone. Our patient makes dramatic recovery after blood transfusion in each presentation.

## Background

Haemoglobin E (HbE)-beta thalassaemia is a one of the commonest forms of thalassaemia in Asian countries [1]. Although HbE may not cause clinical significance, ones it combines with thalassaemia, it will produce variable phenotypes which manifests as moderate to severe anaemia and ineffective erythropoiesis eventually resulting in serious complications. Interestingly, the clinical picture may range from that of beta thalassaemia minor through intermedia to thalassaemia major [2].

Extramedullary hematopoiesis (EMH), a common compensatory phenomenon in thalassemia, is rarely localized to the spinal cord. Spinal cord compression is an extremely rare event in the course of the disease where the hematopoiesis develops intraspinally and neurological manifestations are rarer. Diagnosis is based on history, physical examination and confirmed by magnetic resonance imaging (MRI) findings, and treatment consisted of blood hyper transfusions, surgical resection, radiotherapy alone or combination [3].

## Case presentation

In 2006 November, a 27 year old diagnosed HbE-Beta thalassaemic Sri Lankan Muslim male patient presented to the medical ward with rapidly progressive weakness of both lower limbs for about 1 week duration and ended up in total paralysis of both lower limbs with double incontinence. It did not progress to the upper limbs or respiratory muscles. On examination he had spastic paraparesis with a sensory level at thoracic 8 (T8) level. At that time his haemoglobin was around 5 g/dl and had a number of blood transfusions which settled his paralysis.

In 2007 November, once again he presented to the neurology ward with similar symptoms. Physical examination revealed a young male with a characteristic facies with frontal bossing, prominent malar prominences, short upper limbs and multiple skeletal deformities. He was severely pale and icteric as well. Splenectomy scar was there and liver was moderately enlarged (5 cm below costal margin). Higher functions and cranial nerves examinations were normal. Motor system examination revealed normal upper limbs with increased tone in lower limb muscles and complete loss of power in all muscle groups of the both lower limbs. Deep tendon reflexes were exaggerated in both lower limbs with bilateral extensor planter response. Superficial abdominal reflexes were absent and there was a complete sensory loss below T8 level.

On admission he had haemoglobin of 6.5 g/dl with normal white cell and platelet counts. All the biochemical investigations were normal except high serum bilirubin indirect fraction and very high level of serum ferritin (2488 ng/mL). X ray thoracic spine did not provide any clues of compressive lesions. MRI of the thoracic spine revealed multilevel, lobulated soft tissue mass in the extradural space extending from T5 to T8. The spinal cord and thecal sac was severely compromised with anterior displacement of the cord due to pressure effect on dorsal cord suggesting extra- medullary haemopoiesis.

The patient had undergone number of packed cell transfusions with iron chelating therapy (Intravenous desferioxamine). With blood transfusions the lower extremity power improved gradually and regained sphincter control once his haemoglobin level comes around 10 g/dl. Post transfusion MRI of thoracic spine revealed the reduction of the size of soft tissue mass with minimal pressure effect on the cord, which further strengthening the diagnosis of cord compression due to extramedullary haemopoiesis to overcome the anaemia.

## Discussion

EMH is a compensatory phenomenon in the patients with thalassaemia. Neurologic signs and symptoms are led by epidural masses via direct compression of the nerve roots and the spinal cord. The spinal cord compression with an extra medullary haemopoietic mass in thalassemia was first described by *Gatto* in 1954. It usually affects the lower thoracic spinal cord due to narrow spinal canal [4]. The radiological method of choice for diagnosing extramedullary hematopoietic masses is the MRI of the spine. It characteristically shows an isointense mass with a high spinal intensity rim on T1-weighted images and a hyperintense mass on T2-weighted images [5].

Management of these patients remains controversial. Treatment options for cord compression are surgical removal, radiation therapy or various combinations therapy with blood transfusion to maintain haemoglobin level above 10 g/dl. In emergency cases, intravenous steroids may be used as the temporary measure until definitive treatment is applied. Most reported cases have been treated with surgical decompression with or without radiation therapy or radiation therapy alone [5].

The pathogenesis of EMH may be attributed to the extension of hyperplastic marrow through the thinned out trabeculae related to the proximal end of the ribs. It is also claimed that the remained embryonic cells within the epidural space are transformed into hematopoietic tissue. Multiple blood transfusions would down regulate erythropoietin production. Previous reports have been shown that the improvement is incomplete and effect is short lasting. Therefore it has been used as adjunct therapy to surgery or radiotherapy. However our patient had a dramatic response to hypertransfusion alone. He was advised the necessity of routine haemoglobin measurements and regular transfusions with iron chelation to keep his haemoglobin around 10 g/dl. With the maintenance of haemoglobin 10 g/dl he did not get the recurrences for the last 2 years duration. Therefore hypertransfusion alone seems to be a promising treatment method in patients with EMH.

## Conclusion

Clinical awareness is important for early diagnosis and prevention of irreversible neurologic complications in patients with haemolytic anaemias with EMH. Hypertransfusion seems to be a promising treatment method that should be recommended as a first-line approach with adequate iron chelation therapy rather than subjecting them to invasive procedures or radiation.

## Consent

Written informed consent was obtained from the patient for publication of this Case report and any accompanying images.

## Abbreviations

HbE: haemoglobin E
EMH: extramedullary Haemopoiesis
MRI: magnetic resonance Imaging
T8: thoracic 8
